# Exploring brain network oscillations during seizures in drug-naïve patients with juvenile absence epilepsy

**DOI:** 10.3389/fneur.2024.1340959

**Published:** 2024-03-14

**Authors:** Linjie Tan, Haoling Tang, Hua Luo, Xiu Chen, Zhong Zheng, Jianghai Ruan, Dechou Zhang

**Affiliations:** ^1^Department of Neurology, The Affiliated Hospital of Southwest Medical University, Luzhou, China; ^2^Laboratory of Neurological Diseases and Brain Function, The Affiliated Hospital of Southwest Medical University, Luzhou, China; ^3^Neurobiological Laboratory, West China Hospital, Sichuan University, Chengdu, China; ^4^Center for Neurological Function Test and Neuromodulation, West China Xiamen Hospital, Sichuan University, Xiamen, China; ^5^Department of Neurology, The Affiliated Traditional Chinese Medicine Hospital of Southwest Medical University, Luzhou, China

**Keywords:** juvenile absence epilepsy, electroencephalogram, graph theory, phase lock value, functional connectivity

## Abstract

**Objective:**

We aimed to investigate the brain network activity during seizures in patients with untreated juvenile absence epilepsy.

**Methods:**

Thirty-six juvenile absence epilepsy (JAE) patients with a current high frequency of seizures (more than five seizures during a 2 h EEG examination) were included. Each participant underwent a 2 h video EEG examination. Five 10 s EEG epochs for inter-ictal, pre-ictal, and post-ictal, and five 5 s EEG epochs for ictal states were extracted. Five 10 s resting-state EEG epochs for each participant from a sex- and age-matched healthy control (HC) were enrolled. The topological parameters of the brain networks were calculated using a graph theory analysis.

**Results:**

Compared with the resting state of the HC group, the global efficiency, local efficiency, and clustering coefficients of the JAE group decreased in the inter-ictal state. In addition, the ictal state showed significantly increased global and local efficiency and clustering coefficients (*p* < 0.05) and a decreased small-world index and the shortest path length (*p* < 0.05) in the theta and alpha bands, compared to the remaining states within the JAE group. Moreover, subgroup analysis revealed that those JAE patients with typical 3 Hz discharges had upgraded global efficiency, local efficiency, and clustering coefficients in both delta and beta1 bands, compared to those JAE patients with non-3 Hz discharges during seizures.

**Conclusion:**

The present study supported the idea that the changes in the EEG brain networks in JAE patients are characterized by decreased global and local efficiency and clustering coefficient in the alpha band. Moreover, the onset of seizures is accompanied by excessively enhanced network efficiency. JAE patients with different ictal discharge patterns may have different functional network oscillations.

## 1 Introduction

Juvenile absence epilepsy (JAE) is a common syndrome of idiopathic generalized epilepsy, accounting for 10–17% of children and adolescents (1). JAE usually occurs between the ages of 8 and 20 years and has equal incidence in both sexes. Electroencephalogram (EEG) typically shows a highly recognizable pattern of bilateral, symmetrical, and synchronous 3–4 Hz spike-wave discharges (SWDs) and is characterized by a frequent and brief loss of consciousness or behavioral termination (2–4). JAE is not benign and causes substantial behavioral deficits in many patients with this disorder (2). In addition, studies have shown that JAE often has neuropsychological deficits (5–7). The study of epilepsy has historically involved pathological changes in seizures and during their development. Several studies have reported abnormalities in brain activity and networks primarily during the inter-ictal period in the absence of epilepsy (8) and showed that the inter-ictal period in epileptic patients is characterized by enhanced functional connectivity (FC) networks, changes in a modular structure, prominent hub-like regions, and a shift to a more regular topology (9–12).

Some studies have shown persistent changes in the thalamic and cortical functions in childhood absence epilepsy patients, even in the absence of SWD activity and altered regional homogeneity involving striatal-thalamic-cortical networks (13, 14). In addition, drug naïve childhood absence epilepsy patients undergo significant alterations in resting-state networks of inter-ictal and ictal periods, resulting in dysfunction of specific networks essential for psychosocial functioning (15). However, by now, few studies (16, 17) have been devoted to investigating the topological organization of functional brain networks in JAE patients with typical 3 Hz discharges and atypical discharges during resting state without the confounding effect of antiepileptic drugs and inter-ictal epileptic discharges.

The graph theory analysis methods have been used to investigate the properties of brain networks in patients suffering from mental disorders (18–20). Graph theory was used to analyze the change in patterns of information transmission in the absence of an epilepsy network to help reveal the mechanism of pathological changes before and after the absence of epilepsy. However, only a limited number of studies (10, 21, 22) have used graph-theoretic methods to detect resting cortical functional networks in JAE patients, most of which were magnetoencephalography and functional magnetic resonance data. Few studies, in particular, have investigated the brain networks of JAE patients throughout the seizure period using EEG. EEG is an ideal tool for non-invasive brain activity measurement to identify epileptic foci and capture electrical signals during seizures (23, 24). The absolute value of the average phase difference between two signals of a complex unit length vector is the phase-locked value (PLV), which is a commonly used phase interaction measure, and is now widely used in brain network connectivity analyses to study potential disease abnormalities (25–27). Therefore, we decided to study the changes in the brain networks of JAE patients using EEG-based graph theory analysis and PLV.

In this study, we hypothesized that the onset and termination of absence seizures were accompanied by changes in the functional networks of the brain. We aimed to investigate how functional brain networks change in JAE patients in four states, namely, pre-ictal, ictal, post-ictal, and inter-ictal states. In addition, EEG-ROI (28) functional connectivity analyses were performed.

## 2 Materials and methods

### 2.1 Participants

Thirty-six drug-naïve patients with JAE were recruited from January 2016 to January 2022 in the department of neurology at the affiliated hospital of Southwest Medical University. The diagnoses of absence epilepsy were established according to the recommendations of the International League Against Epilepsy (29). The participants were included according to the inclusion criteria as follows: to be enrolled, JAE patients had to be right-handed, present 2.5–4 Hz SWDs on EEG with a high frequency of absence seizures and more than five episodes of seizures within the 2 h of video EEG examination, absence epilepsy without convulsions, no anatomical abnormalities were observed on cranial MRI, and antiepileptic medications had never been taken. The participants were excluded according to the exclusion criteria as follows: patients with a history of seizures other than absence seizures, such as myoclonic seizures; patients with a previous history of other major neuropsychiatric disorders; patients with poor quality of EEG data; the patient was in critical condition or unconscious; and patients who were non-compliant. Right-handed, healthy control subjects matched for age and sex were included in the study with no medical or family history of neuropsychiatric disorders who visited our hospital for routine physical examinations. Based on the inclusion and exclusion criteria, 36 patients with JAE (age 11.33 ± 5.26 years) and 36 age- and sex-matched healthy controls (HCs) (age 12.83 ± 3.26 years) were included in this study. No significant differences in age or sex between the two groups were found (Table 1). The study was approved by the Ethics Committee of the Affiliated Hospital of Southwest Medical University. Written informed consent was obtained from the included participants. If patients were less than 18 years of age, their legal guardians provided informed consent.

**Table 1:** The sex and age of participants in this study.

	JAE (*n* = 36)	HC (*n* = 36)	*χ*^2^/t	*p*-value
Sex (male/female)^a^	21/15	22/14	0.058	0.810
Age (years, mean ± SD)^b^	11.33 ± 5.26	12.83 ± 3.26	−1.453	0.152

a Chi-square test.

b Two sample *t*-test; JAE, juvenile absence epilepsy; HC, healthy control.

### 2.2 EEG data acquisition and processing

#### 2.2.1 Data acquisition

All participants were asked to relax and keep their eyes closed, and their scalp EEG was recorded in a quiet and relaxed state. Video EEGs were continuously recorded using a 19-channel analog recorder (Galileo EB Neuro with a camera) for 2 h according to the international 10–20 system. Video EEG data were recorded in a time-locked pattern. The impedance of each electrode was kept at less than 10 kΩ. The sampling rate was 500 Hz. The leads (Fp1, Fp2, F3, F4, F7, F8, T3/T7, T4/T8, T5/P7, T6/P8, C3, C4, P3, P4, O1, O2, Fz, Cz, and Pz) were placed using a quantified ruler. After data acquisition, two experienced clinicians independently reviewed, and the entire dataset included subjects with 2.5–4 Hz SWDs. Furthermore, 2 h of video EEG data were obtained from 36 age- and sex-matched HCs.

#### 2.2.2 Data preprocessing

EEG data preprocessing was similar to previous studies (30, 31). The rough approach was to export the raw data in the European Data Format (EDF). After further FIR filtering, the EEG was bandpass filtered from 1 to 45 Hz using the Hamming window. Electromyography artifacts and muscle artifacts were then completely removed using the EEGlab plug-in in AAR.^1^ The EEG matrix with a low fractal dimension would be identified as electrooculogram components and removed automatically. Then, the EEG was recomputed to the common average reference. After preprocessing, the EEG of interest was cut out and translated to text format for further analysis. According to our experience and prior research (32), EEG analysis parameters exhibit stability and can be reliably estimated when the length of resting-state EEG for each participant is larger than 30 s. EEG data with a duration of 50 s per participant or condition can yield consistently stable results.

We extracted five 10 s epoch EEG data for the patients for inter-ictal, pre-ictal, and post-ictal conditions. We obtained five 5 s epoch EEG data in the seizure. The EEG data were preprocessed in the EEGLAB (33) (v14.1.1, http://sccn.ucsd.edu), which operates in the MATLAB (R2016b, MathWorks, Inc.) environment.

### 2.3 Construction of the PLV network matrix

The PLV algorithm has been described in previous studies (27, 34, 35). In brief, the bandpass-filtered signals $s_{x}\left(t\right)$ were transferred using Hilbert transformation to determine the instantaneous phase of EEG signals ${\tilde{s}}_{x}\left(t\right)$. Then, the signals can be represented as Equation (1):


(1) $$z_{x}\left(t\right)=s_{x}\left(t\right)+j{\tilde{s}}_{x}\left(t\right)=A_{x}\left(t\right)e^{j\emptyset_{x}\left(t\right)},$$

where $A_{x}$ denotes the instantaneous amplitude and $\emptyset_{x}\left(t\right)$ indicates the instantaneous phase. Finally, PLV between two signal series can be calculated with the following Equation (2):


(2) $${\mathrm{PLV}}_{t}=\frac{1}{N}|\overset{N}{\underset{n=1}{\sum }}\exp\left(j\theta \left(t,n\right)\right)|,$$

where θ(t,n) is the phase difference between two signals $s_{x}\left(t\right)$ and $s_{y}\left(t\right)$ at time *t,* and *N* is the number of signal segments.

PLV, depicting the absolute value of the mean phase difference between signals, serves as a metric to evaluate the level of synchronization among EEG signals within a specific frequency band. According to graph theory, an effective exchange of information is possible when signals are in a locked-in relationship. The PLV takes values on [0, 1]. A 0 reflects the case where there is no phase synchronization and the two signals do not have apparent synchronization, and 1 reflects the case where the extent of synchronization between the two signals is the strongest (36). In the present study, five frequency bands were defined: delta band (1–4 Hz), theta band (4–8 Hz), alpha band (8–13 Hz), beta1 band (13–30 Hz), and beta2 band (30–45 Hz). Then, a 19 × 19 PLV matrix was constructed for each subject and used for later graph theory analysis.

### 2.4 Computation of graph metrics

This study used the 19 EEG channels as nodes for each subject in each frequency band. The previously constructed 19 × 19 PLV matrix was used for graph theory analysis. Then, we computed the following measures: clustering coefficient, local efficiency, global efficiency, shortest path length, and small-world index (37–39). The shortest path length represents the minimum number of edges that must be traversed to get from one node to another (40) and is calculated as the average of the shortest paths between all pairs of nodes. The shortest path length is the opposite of the clustering coefficient, which reflects the overall efficiency of information integration between different brain regions. The cluster coefficient refers to the degree to which two adjacent nodes are connected to each other. The clustering coefficient is generally considered to be an indicator of the efficiency of information processing in the local brain region of the brain network. The global efficiency referred to the reciprocal average of the shortest path length, which represents the efficiency of signal transmission between network nodes, and the local efficiency was the trend to measure the clustering of nodes with strong connections in a network. These graph-based indices were computed using MATLAB functions embedded in the brain connectivity toolbox.^2^

### 2.5 Source location based on functional network analysis

To further explore the functional network changes in peri-seizures, a source-based FC analysis was performed. Eighty-four Brodmann areas (BAs) were defined as regions of interest (ROIs) (Supplementary Table S1). We extracted the EEG signal of the 84 ROIs by using the exact low-resolution electromagnetic tomography inverse algorithm (eLORETA) (41). The eLORETA method is used to locate scalp EEG signals from multiple distributed three-dimensional cortical sources (42). It has been applied in a variety of EEG-related studies (30, 43, 44). Detailed mathematical algorithms were shown in previous studies (41, 42). These operations were implemented on LORETA-KEY (www.uzh.ch/keyinst/loreta, the brain-heart: KEY Institute in Zurich, Switzerland) analysis software. After the signals of each ROI were extracted, the PLV matrices and graph parameters, including clustering coefficients, shortest path lengths, small-world parameters, global efficiency, and local efficiency, were calculated using the above described methods.

### 2.6 Sub-group analysis

The JAE group was divided into subgroups according to sex, age, and discharge patterns. The male group consisted of 21 patients, and the female group consisted of 15 patients. Two subgroups were obtained and were divided by age: 19 in the young group (age less than or equal to 13 years) and 17 in the elder group (age more than 13 years). In addition, subgroups of typical 3 Hz discharges (*n* = 21) and atypical discharges (*n* = 15) were classified.

### 2.7 Statistical analysis

The measured age of the participants was represented by mean ± standard deviation (SD), and the sex was represented by ratios. A generalized linear model (GLM) with sex and age as covariates was employed to eliminate the possible effects in the comparisons of FC and network properties between AE inter-ictal and HC rest states. Repeated measure ANOVA was used to compare the overall differences between the PLV and graph parameters for the four subgroups within the AE group. *Post hoc* comparisons for the four conditions whining AE group were performed using the Tukey-Kramer method. All the tests were conducted using MATLAB (R2016b, The MathWorks Inc.).

## 3 Results

### 3.1 Comparisons of brain FC between inter-ictal of JAE and HC rest states

**Figure 1:**
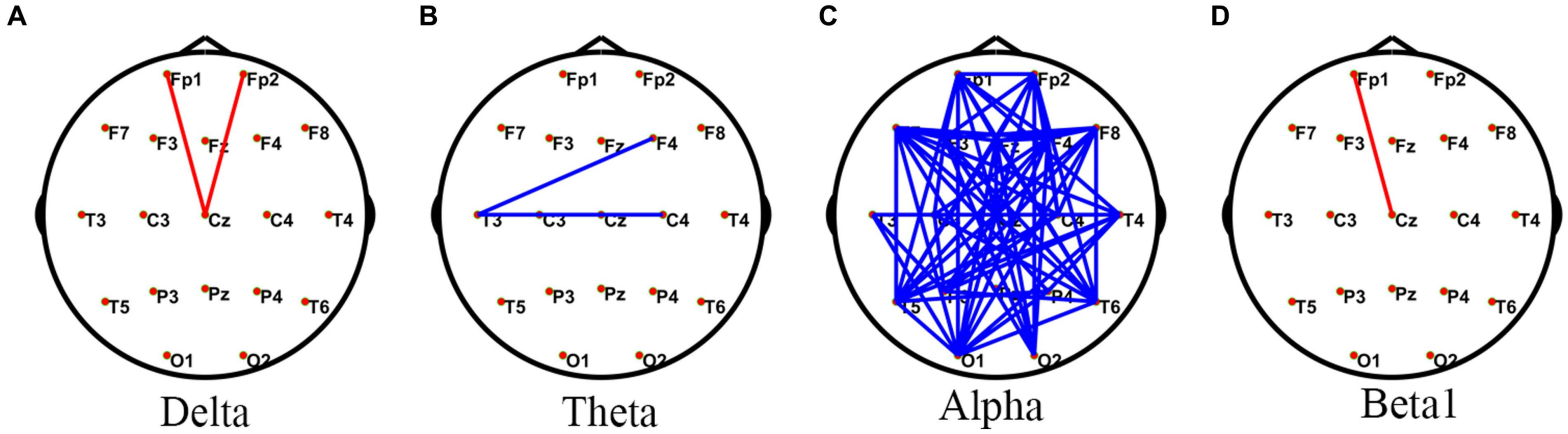
Comparisons of PLV network between JAE and HC groups. ^*^PLV network connectivity in the delta (1–4 Hz) **(A)**, theta (4–8 Hz) **(B)**, alpha (8–13 Hz) **(C)**, and beta1 (13–30 Hz) **(D)** bands. The red line in the figure indicates increased FC in JAE patients compared to HC after two sample *t-*test with FDR correction (FDR *p* < 0.05). The blue line in the figure indicates that FC is reduced in JAE patients compared to the HC (FDR *p* < 0.05). PLV, phase locked values; FC, functional connectivity; JAE, juvenile absence epilepsy; HC, healthy control.

Compared with the HC rest state, JAE patients had a small number of edges with enhanced connectivity between the forehead and central regions in the delta and beta1 frequency bands, while in the alpha frequency band, JAE patients had a significant number of edges with reduced FC between the left frontal lobe and the right posterior temporal and occipital areas, as well as between the right frontal area and left posterior temporal and occipital regions (Figure 1). Source localization based on FC analysis showed a reduction in FC involving extensive connections in the alpha band during the inter-ictal period in JAE compared to the HC group (Supplementary Figure S1). Compared with the HC group, the mean intra-network FC involving parietal, limbic, and sub-lobar networks decreased in the alpha band (Supplementary Figure S2). For the mean inter-network FC, JAE patients showed upgraded FC involving frontal and occipital, sub-lobar networks, occipital and sub-lobar networks, and temporal and limbic networks in the delta band (Supplementary Figure S2). In addition, a large number of inter-network FC decreased in the alpha band of JAE patients (Supplementary Figure S2).

### 3.2 Comparisons of brain FC within the JAE group

**Figure 2:**
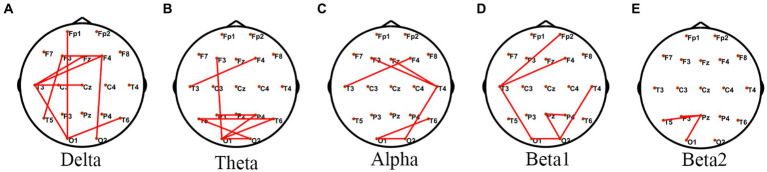
Comparisons of PLV networks within the JAE group using one-way ANOVA. ^*^PLV network connectivity in the delta (1–4 Hz) **(A)**, theta (4–8 Hz) **(B)**, alpha (8–13 Hz) **(C)**, beta1 (13–30 Hz) **(D)**, and beta2 (30–45 Hz) **(E)** bands. The red lines in the figure indicate significant differences in these edges.

Comparison of PLV within the JAE group revealed differences in functional connectivity in the frontal, temporal, and occipital lobes in the delta, theta, alpha, and beta bands (Figure 2). Based on 84 ROIs functional network analysis, the frontal, temporal, and sub-lobar lobes showed significant differences in FC in the delta, theta, alpha, and beta frequency bands (Supplementary Figure S1). *Post hoc* analysis showed that compared with the pre-ictal state, the FC networks among frontal, parietal, temporal, limbic, and sub-lobar networks increased in the delta, theta, alpha, and beta1 bands of the ictal state. Similar FC change patterns were found when comparing the ictal state to post-ictal and inter-ictal states. Compared with the inter-ictal state, the post-ictal state showed enhanced FC among frontal, temporal, and sub-lobar networks in the delta band. In the pre-ictal state, the FC between frontal and sub-lobar, limbic, temporal, occipital, and parietal lobes increased in the delta band when compared with those of the inter-ictal state (Supplementary Figure S3). When we were calculating the mean intra-network and mean inter-network FC, the main finding was that numerous intra-network FC of subnetworks in the delta, theta, alpha, and beta1 bands increased in the ictal-state within the JAE group (Supplementary Figure S4). Moreover, the inter-network FC involving a large number of connections between sub-networks in the delta, theta, alpha, and beta1 bands significantly upgraded in the ictal-state, compared with other states within JAE patients (Supplementary Figure S5).

### 3.3 Decreased network efficiency in inter-ictal of JAE

**Figure 3:**
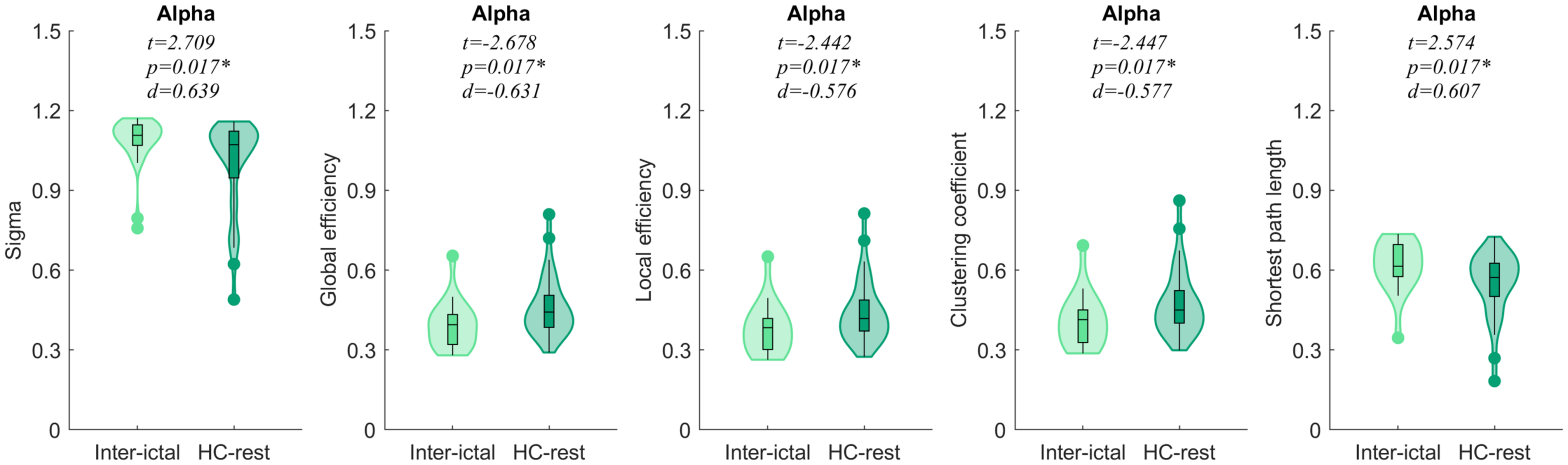
Comparisons of network properties between inter-ictal of the JAE group and the HC-rest group^#^. ^#^Generalized linear models (GLM) with age and sex as covariates were used to eliminate their possible artifacts on the results. ^*^p < 0.05 after FDR correction. Sigma, small-world index.

Inter-ictal of JAE manifested a higher small-world index and the shortest path length in the alpha frequency bands than HCs, while the global efficiency, local efficiency, and clustering coefficient were lower, and the difference was statistically significant (*p* < 0.05) (Figure 3). No significant difference in the remaining frequency bands was found between the JAE and the corresponding HC rest group. Based on 84 ROI analyses, the differences were also mainly observed in the alpha (8–13 Hz) band. Compared to the HC rest group, the inter-ictal of JAE has increased the small-world index and the shortest path lengths and decreased global efficiency, local efficiency, and clustering coefficients (*p* < 0.05). In the delta and theta bands, the local efficiency and clustering coefficients were increased in the inter-ictal of JAE patients compared to the HC-rest group (*p* < 0.05) (Supplementary Figure S6).

### 3.4 Excessively enhanced network efficiency during ictal states

**Figure 4:**
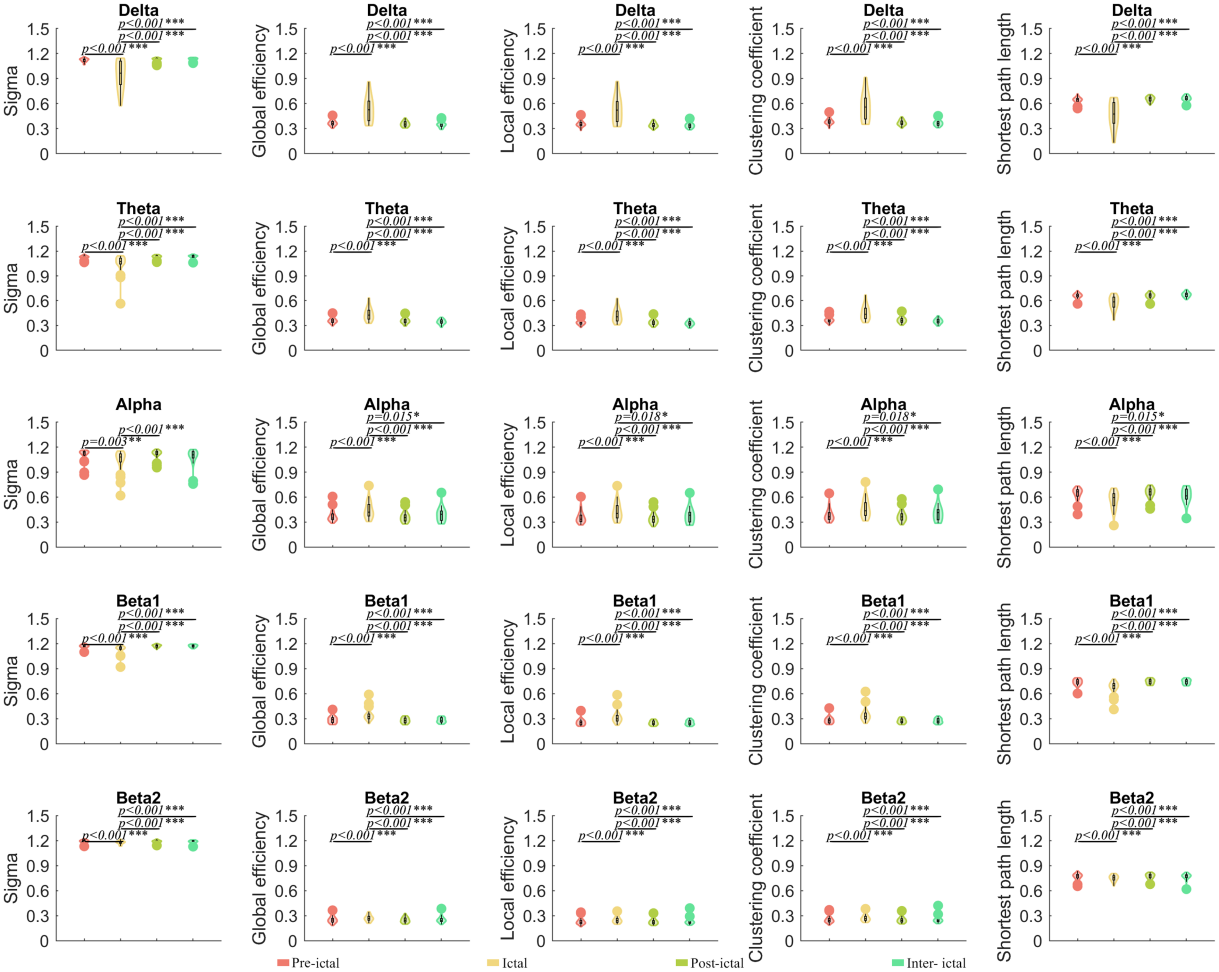
Comparisons of network properties within the JAE group^#^. ^#^Global efficiency, local efficiency, and clustering coefficients in the delta, theta, alpha, and beta frequency bands were significantly lower in the pre-ictal, post-ictal, and inter-ictal periods, compared to the ictal period, while small-world index and shortest path lengths were instead elevated. Repeated measure ANOVA was used to compare the overall differences. The Tukey–Kramer method was used in the *post hoc* paired comparisons. ^*^p < 0.05, ^**^p < 0.01, and ^***^p < 0.001. Sigma, small-world index. The legends are shown below the figure.

Compared to the ictal periods, the pre-ictal, post-ictal, and inter-ictal periods had significantly lower global efficiency, local efficiency, and clustering coefficients in the delta frequency band (*p* < 0.05), while the small-world index and the shortest path length were instead higher (*p* < 0.05). The same changes could be observed in the theta, alpha, and beta frequency bands (Figure 4). A total of 84 ROI analyses showed that ictal has higher global efficiency, local efficiency, and clustering coefficients (*p* < 0.05) and a lower small-world index and the shortest path lengths (*p* < 0.05) in the theta and alpha bands compared to the remaining three states (Supplementary Figure S7). There were no significant differences in parameters between the three states of pre-ictal, post-ictal, and inter-ictal periods.

### 3.5 Heterogeneous network properties between sub-groups with different discharge characteristics

**Figure 5:**
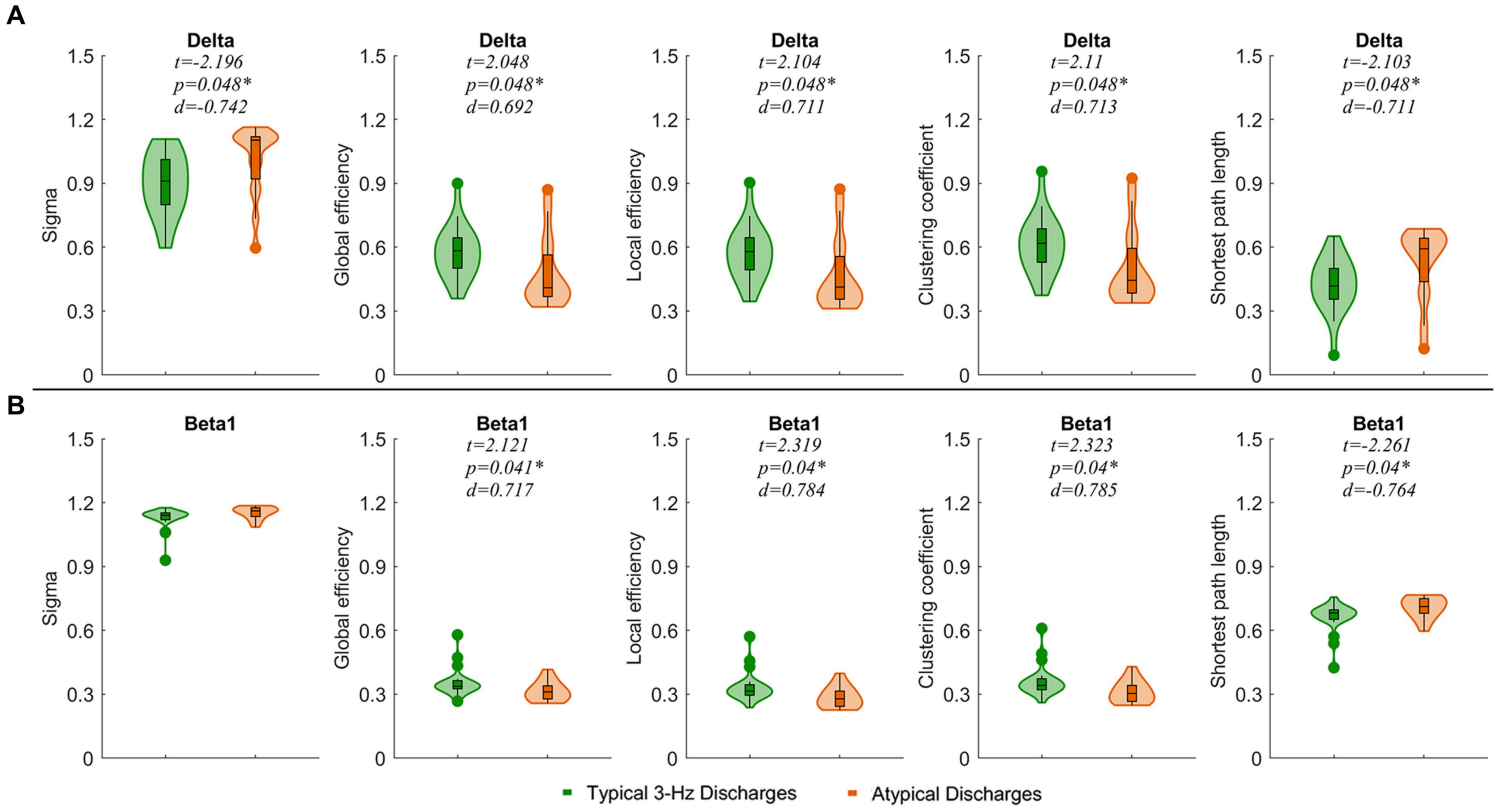
Comparisons of network properties between ictal of typical 3 Hz discharges and atypical discharge in the delta (1–4 Hz) **(A)** and beta1 (13–30 Hz) **(B)** bands. ^*^p < 0.05 after FDR correction, Sigma, small-world index. The legends are shown below the figure.

We observed that typical 3 Hz discharges showed significantly decreased small-world indices and the shortest path lengths and increased global efficiency, local efficiency, and clustering coefficient compared to atypical discharges in the delta band (*p* < 0.05). In the beta1 band, the local efficiency, global efficiency, and clustering coefficient of typical 3 Hz discharges were significantly enhanced (*p* < 0.05), and the shortest path length was weakened compared to atypical discharges (Figure 5).

## 4 Discussion

This study investigated the functional network characteristics of JAE during per-ictal using graph theory analysis based on the scalp. We confirm that brain networks undergo changes in patients with JAE, characterized by decreased clustering coefficients, global efficiency, and local efficiency and an increased small-world index and the shortest path length. These discoveries have deepened our comprehension of JAE in terms of brain electrophysiology. PLV brain network analysis revealed significant differences in network connectivity between inter-ictal of JAE patients and HC, especially in the alpha band, and these differences were basically concentrated in the temporal, parietal, and occipital regions, which were manifested as weakened connectivity in the region. In the delta and beta1 bands, the JAE group had enhanced connectivity in the frontal-parietal lobe compared to the healthy control group. It has been suggested that changes in PLV values in this band are related to cognitive function, so we speculate that changes in the beta1 band functional connectivity in JAE patients may be related to their persistent cognitive dysfunction (45, 46).

The study analyzed four states of JAE episodes from a brain network perspective, and small amounts of limbic enhancement were found in frontal, parietal, and occipital regions in almost all frequency bands in all four states. Previous EEG-fMRI studies found enhanced connectivity in the sensorimotor network before JAE episodes, involving prefrontal and precuneus regions (47). This suggests that sensorimotor networks may be involved in the initiation process of SWD. The main changes from pre-ictal to post-ictal to inter-ictal periods are a gradual increase in brain synchrony and a gradual generalization of brain areas involved in the increased synchrony. This is consistent with the previous study that cortico-cortical networks play a crucial role in the initiation and maintenance of SWD. From the point of view of the structural foundation, the seizure process is a gradual process that may involve the cortico-thalamic-cortical circuits, especially the frontal region (48, 49). The network system composed of the frontoparietal temporal cortex, such as the medial frontal lobe, anterior and posterior cingulate gyrus, central parietal lobe, lateral frontal lobe, orbitofrontal lobe, lateral temporoparietal junction area, and thalamus, is thought to be related to attention and executive function (50), suggesting that the changes in EEG connections in this network may be related to cognitive impairment in children with JAE, especially attention and executive functions. This is also the most common cognitive deficit in children with JAE (51).

In addition, the neural activity networks and information processing in the brain can be explained from graph theory-based network attribute analysis. In the present study, we found abnormal changes in the inter-ictal period of JAE patients in the alpha frequency band compared with the normal group, as evidenced by reduced global efficiency, local efficiency, and clustering coefficients. This is consistent with previous findings (52, 53), which suggest that the efficiency or “speed” of information transfer among the adjacent nodes and the density of the local interconnectivity within a network is compromised. However, inter-ictal JAE patients have a higher small-world index and the shortest path length compared to the HC group. Notably, a higher shortest path length and small-world index are thought to be associated with better cognitive performance (54). Although no neuropsychological test was performed in the current study, cognitive deficiencies associated with JAE have been observed in previous studies. JAE patients have less efficient organization of functional brain networks, so it is reasonable to suggest that there may be a relationship between impaired neurocognitive performance and global functional brain network efficiency.

Previous studies have suggested that epileptiform discharges may occur before seizures (55). In EEG-fMRI studies, patients with JAE have partially activated brain areas in the pre-ictal state (56, 57). In the within-group comparison of the JAE, we only found significantly higher global efficiency, local efficiency, and clustering coefficients during the ictal period compared to the pre-ictal, post-ictal, and inter-ictal periods. Unfortunately, no significant differences were found between the remaining three states. A study has shown that the dynamics of the EEG change before and after a seizure in people with JAE. That is, patients with JAE “slow down” the transition between EEG microstates before and after a seizure (58). This may be due to the ability of EEG microstate analysis to segment EEG signals of spontaneous EEG activity with millisecond temporal resolution and to describe the organizational and temporal dynamics of large-scale cortical oscillations.

Typical absence seizures are more common and are characterized by a loss of consciousness and extensive 3 Hz SWDs on the EEG during the same period. Typical absence epilepsy shows only mild or no cognitive impairment, while patients with atypical absence seizures often have severe cognitive impairment (59). However, in subgroup analyses, graph-theoretic parameters for typical 3 Hz discharges showed increased global efficiency, local efficiency, and clustering coefficients and attenuated small-world indices and shortest path lengths. Some studies claim that a higher small-world index and the shortest path length are thought to be associated with better cognitive performance (54, 60), which is the opposite of our results. This may be due to these studies using fMRI, not EEG in this study. The spatial resolution of EEG is low, in contrast to functional magnetic resonance imaging, which has a higher spatial resolution. The network properties of AE may be different in different scales.

There are some limitations to this study. This study did not explore the relationship between JAE brain networks and clinical characteristics, such as their relationships with disease prognosis. A longitudinal cohort follow-up study with detailed cognitive domain-assessed observation is needed to overcome these limitations.

## 5 Conclusion

Our study confirmed the notion that the altered brain networks exist in the resting state of JAE patients, and the changes in EEG brain networks in JAE patients are characterized by decreased global efficiency, local efficiency, and clustering coefficient in the alpha band. Moreover, the onset of seizures is accompanied by excessively enhanced network efficiency. JAE patients with different ictal discharge patterns may have different functional network oscillations. Our study provided a novel understanding of the seizure onset mechanisms of JAE from an electrophysiological view and may be applied in the neural regulation treatment of JAE in the future.

## Data availability statement

The raw data supporting the conclusions of this article will be made available by the authors, without undue reservation.

## Ethics statement

The studies involving humans were approved by Ethics Committee of the Affiliated Hospital of Southwest Medical University. The studies were conducted in accordance with the local legislation and institutional requirements. Written informed consent for participation in this study was provided by the participants’ legal guardians/next of kin.

## Author contributions

LT: Investigation, Data curation, Writing – original draft. HT: Data curation, Investigation, Writing – original draft. HL: Formal analysis, Supervision, Validation, Writing – review & editing. XC: Formal analysis, Supervision, Validation, Writing – review & editing. ZZ: Supervision, Writing – review & editing. JR: Supervision, Writing – review & editing, Conceptualization, Formal analysis, Funding acquisition, Investigation, Methodology, Resources, Software, Validation, Visualization. DZ: Formal analysis, Software, Supervision, Writing – review & editing.

## Footnotes

^1^ http://germangh.com

^2^ http://www.brain-connectivity-toolbox.net
