# Supplementary material for: Exploring brain network oscillations during seizures in drug-naïve patients with juvenile absence epilepsy
Source: Front Neurol. 2024 Mar 14;15:1340959. doi: 10.3389/fneur.2024.1340959 (PMC10972980; doi:10.3389/fneur.2024.1340959)
Supplement: Supplementary file 1 [file Data_Sheet_1.docx]

**Appendix 1:**

**Exploring Brain Network Oscillations during Seizures in Drug-Naïve Patients with Juvenile Absence Epilepsy**

Linjie Tan^1,2^, Haoling Tang^1,2^, Hua Luo^1,2^, Xiu Chen^1,2^, Zhong Zheng^3, 4^ , Jianghai Ruan ^1,2^*, Dechou Zhang^5^

**Appendix1**

**Methods to compute of graph metrics**

In this study, we computed the following measures: clustering coefficient (C), local efficiency (Elocal), global efficiency (Eglobal), shortest path length (Lp), and small-world index(sigma). The following formulas were used to compute these parameters.

**Clustering coefficient (C):**

$$C_{i}=\frac{2E_{i}}{K_{i}(K_{i}-1)}$$

The clustering coefficient, denoted as C(i) for node i, depicts the ratio between the actual number of edges and the total possible number of fully connected edges within the sub-network centered around node i. Ei is the actual number of edges for node i, ki is the numbers of neighbor nodes for node i. Then, the clustering coefficient C of the graph is computed by calculating the average of Ci with following formula:

$$C=\frac{1}{N}\sum_{i=1}^{N} C_{i}$$

**The shortest path length**

$$L_{P}=\sum_{i\geq j} \frac{L_{ij}}{\frac{1}{2}N(N+1)}$$

The shortest path length is depicted by L, L_ij_ indicated the shortest distance from node i to node j refering to how many times the connection from node i can reach node j. The average of the distances of all nodes is the average shortest path length (Lp) of the entire network. N is the number of nodes in the network.

**Global efficiency**

$$E_{global}=\frac{1}{N(N-1)}\sum_{i\neq j} \frac{1}{L_{ij}}$$

Global efficiency (E_global_) characterizes the overall effectiveness of information transmission within the graph and can be calculated as the average reciprocal of the shortest paths across all nodes in the network

**Local efficiency**

$$E_{local}=\frac{1}{N}\sum_{i\in G} E_{global}$$

Local efficiency (E_local_) is calculated as the average reciprocal of the shortest paths among all nodes in the network.

**Small-world index (Sigma)**

$$Sigma=\frac{\frac{C_{real}}{C_{random}}}{\frac{L_{real}}{L_{random}}}$$

The small-world index Sigma is calculated by using the value of clustering coefficient relative to a random network divided by the shortest path length relative to a random network. when Sigma > 1, the network has the “small-world” property.

S.Table1 The regions of interest (ROIs) defined by Brodmann areas in the source based functional network analysis.

| Brain Cortex | Bilateral Brodmann Areas |
| --- | --- |
| Frontal Lobe (22 ROIs) | 4, 6,8,9,10,11,13,44,45,46,47 |
| Parietal Lobe (16 ROIs) | 1,2,3,5,7,39,40,43 |
| Occipital Lobe (6 ROIs) | 17,18,19 |
| Temporal Lobe (18 ROIs) | 20,21,22,27,28,37,38,41,42 |
| Limbic Lobe (12 ROIs) | 31,32,33,34,35,36 |
| Sub-lobar (10 ROIs) | 23,24,25,29,30 |


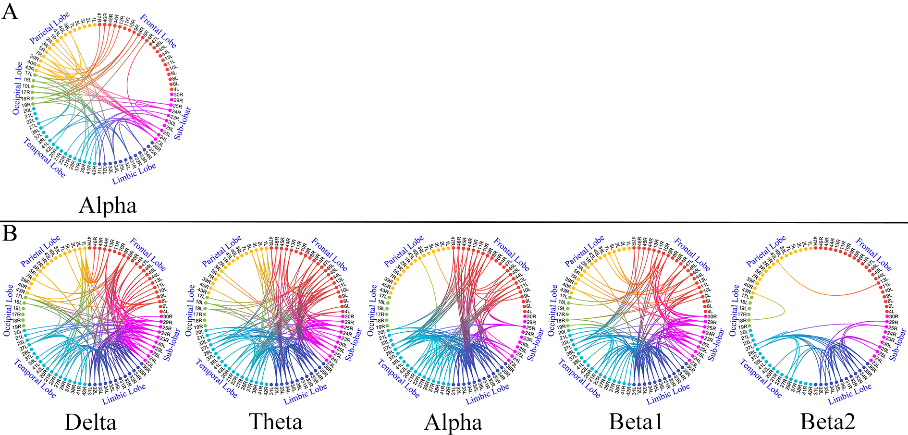


S.Fig.1 FC comparisons based on 84 regions of interest ^#^. A Enhanced HC FC networks compared to inter-ictal state in JAE group. B ANOVA test based on the FC of 84 regions of interest within JAE group. ^#^ two sample *t* test with FDR correction (FDR *p*<0.05) were used in the comparisons between resting state of HC group and inter-ictal state of JAE group. In the ANOVA test, Repeated measure ANOVA was used to compare the overall differences between the conditions within JAE patients. All the connections with *p*<0.05 were shown in the figures.


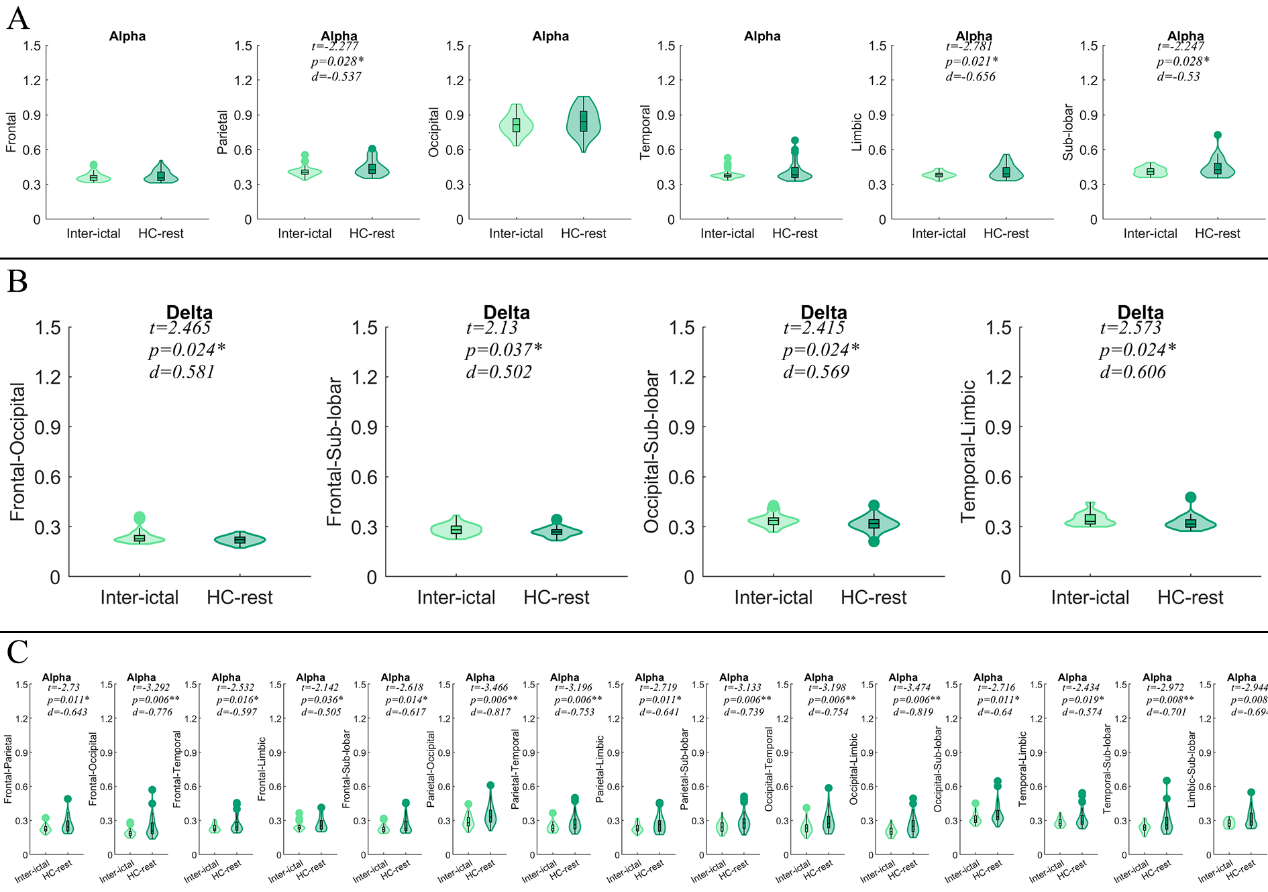


S.Fig.2 Comparisons of mean intra- and inter- network FC of sub-networks. A Comparisons of the mean intra-network FC in alpha band between inter-ictal of AE group and resting state of HC group. B Comparisons of the mean inter-network FC in delta band between inter-ictal of AE group and resting state of HC group. C Comparisons of the mean inter-network FC in alpha band between inter-ictal of AE group and resting state of HC group. **P* < 0.05, ***P* < 0.01, ****P* < 0.001.


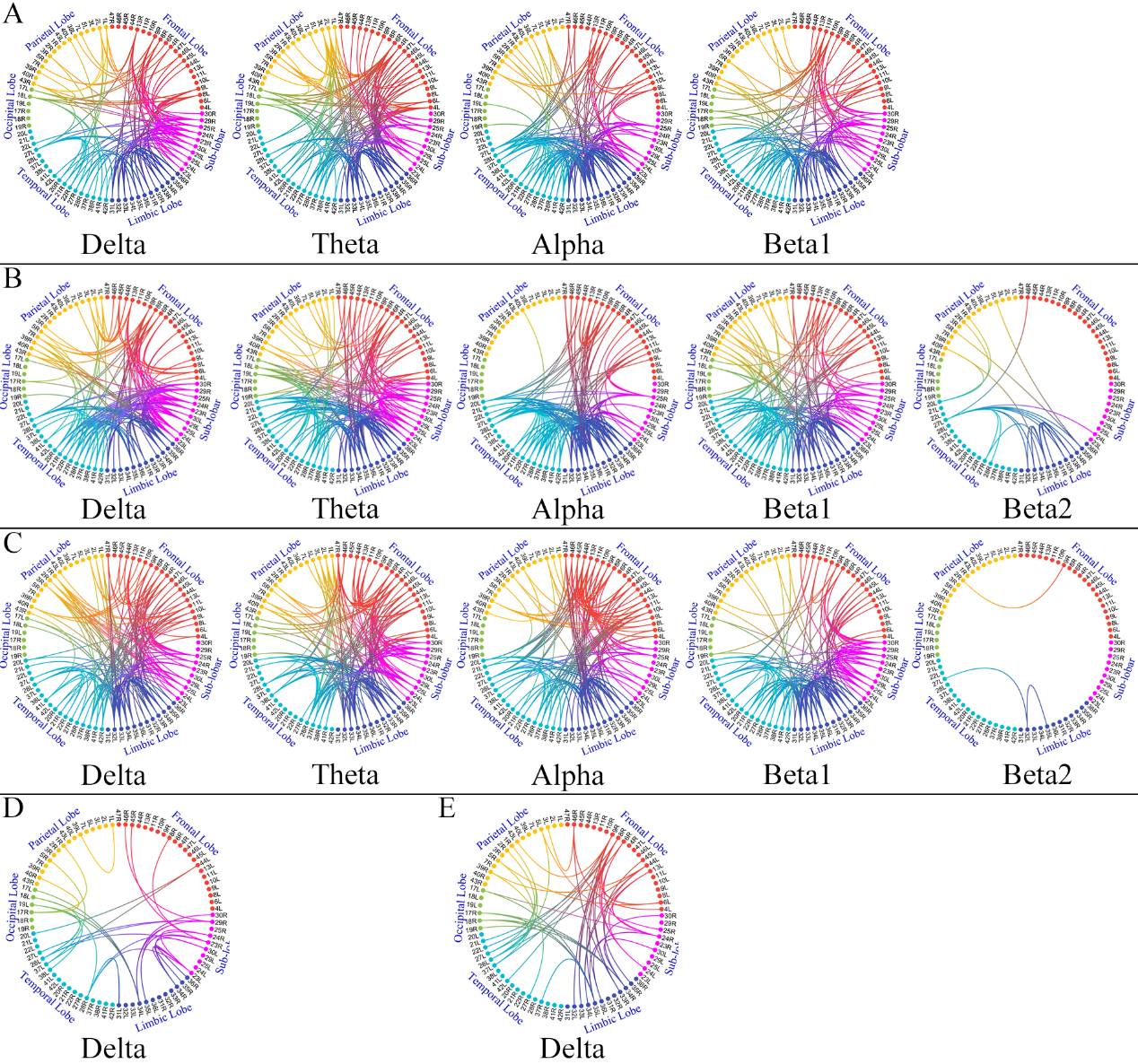


S.Fig.2 FC networks based on 84ROI^#^. ^#^**(A)**Enhanced ictal FC networks compared to pre-ictal, **(B)**ictal FC networks enhanced compared to inter-ictal, **(C)**ictal FC networks enhanced compared to post-ictal, **(D)**enhanced post-ictal FC networks compared with inter-ictal, **(E)** pre-ictal FC networks enhanced compared to inter-ictal. ROI, region of interest; FC, functional connectivity; HC, healthy control.


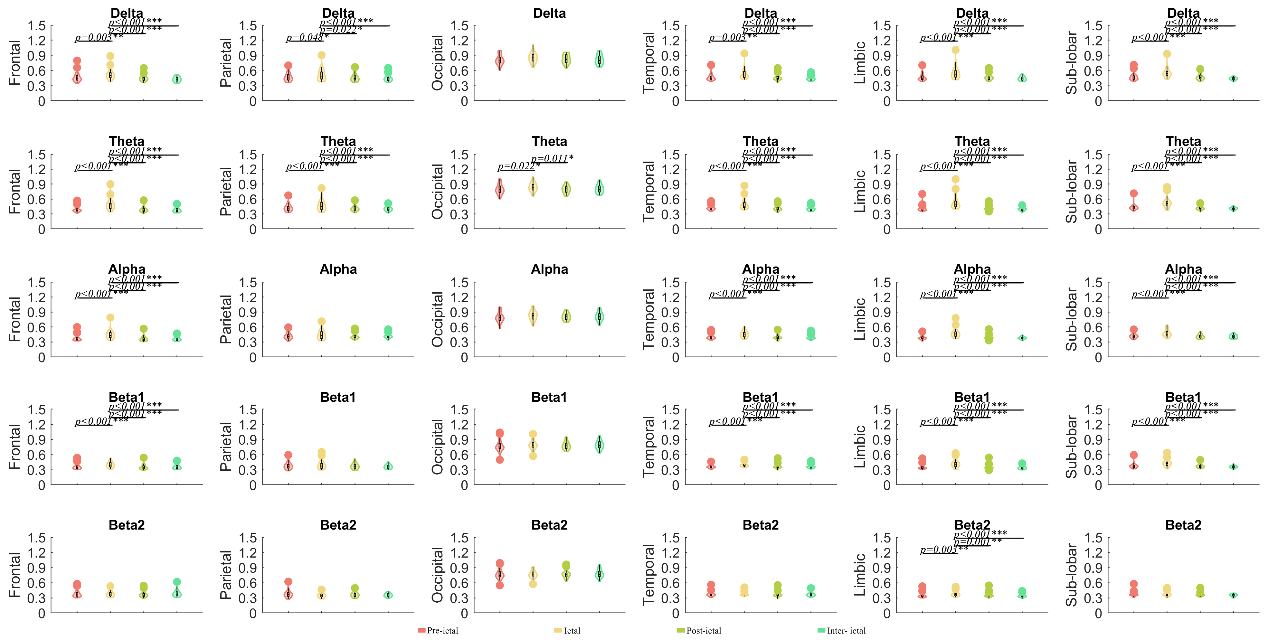


S.Fig.4 Comparisons of mean intra-network FC of sub-networks within AE group in delta, theta, alpha, beta1 and beta2 bands^#^. ^#^The legends were shown below the figure. **P* < 0.05, ***P* < 0.01, ****P* < 0.001.


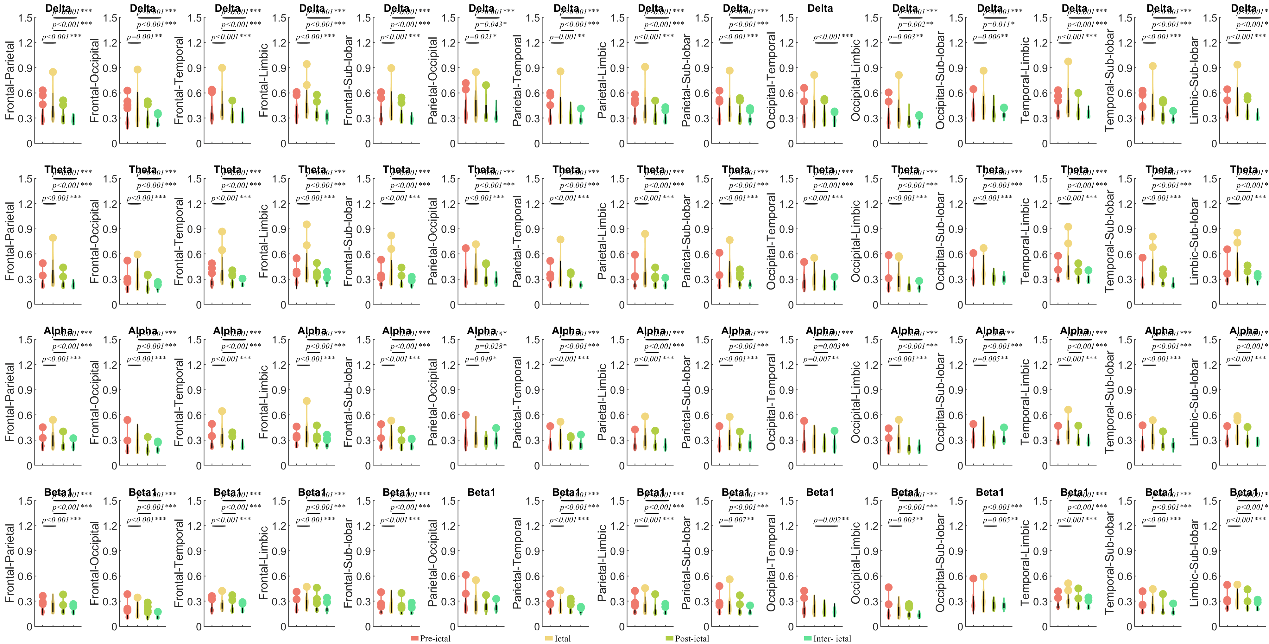


S.Fig.5 Comparisons of mean inter-network FC of sub-networks within AE group in delta, theta, alpha and beta1 bands^#^.

^#^In beta2 bands, the four conditions within AE showed no significant differences. The legends were shown below the figure. **P* < 0.05, ***P* < 0.01, ****P* < 0.001.


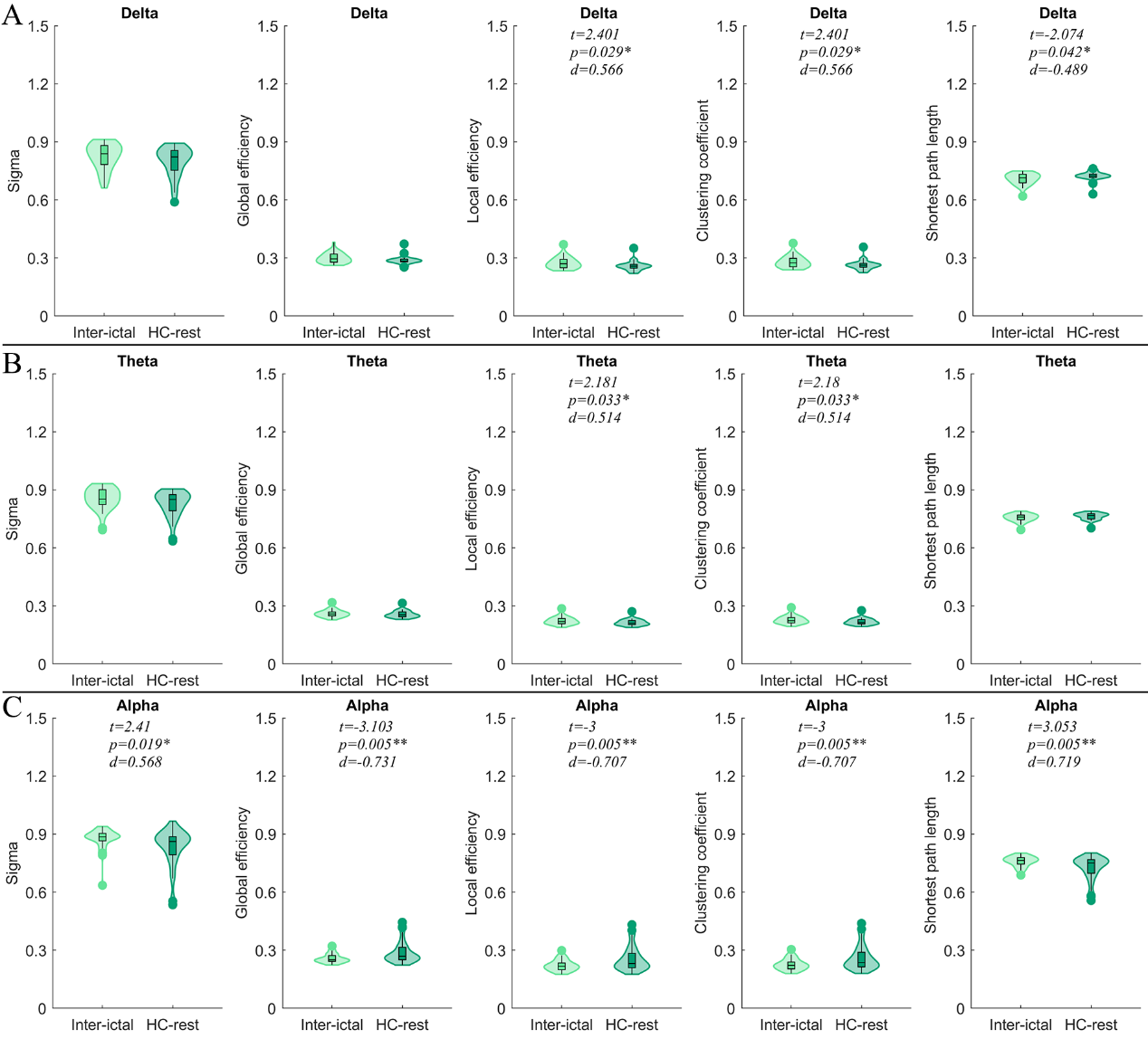


S.Fig.6 Results of network parameters based on 84 regions of interest between inter-ictal of JAE and HC group#. #**(A-B)** The local efficiencies and clustering coefficients were higher in the delta and theta bands in inter-ictal compared to the HC group. **(C)** Compared to HC-rest, the small-world and shortest path lengths of the inter-ictal increase, and the global efficiency, local efficiency, and clustering coefficients decrease in the alpha band. **P* < 0.05, ***P* < 0.01. The legends were shown below the figure.


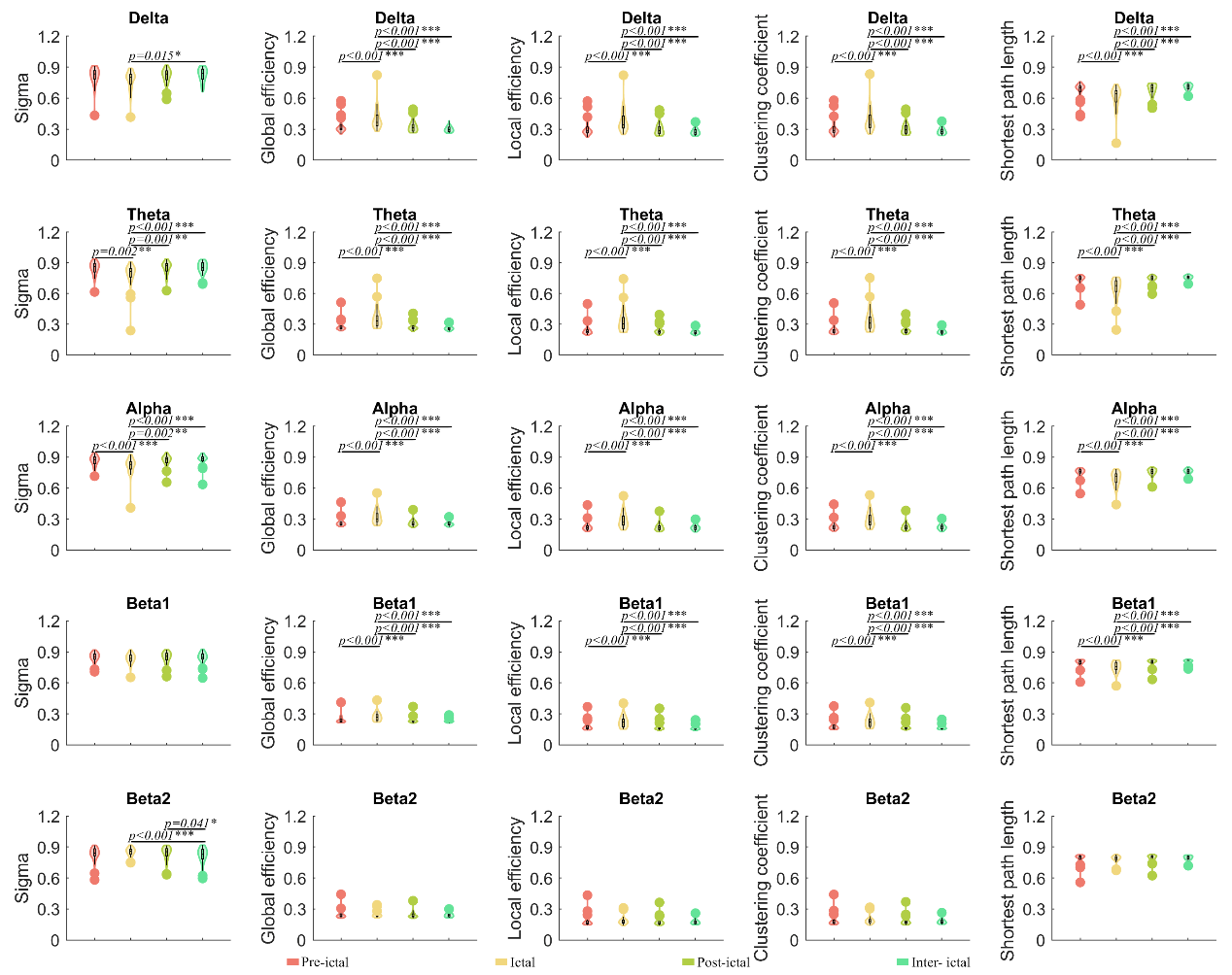
S.Fig.7 Results of network parameters based on 84region of interest within JAE group#. #The ictal period has higher global efficiency, local efficiency, and clustering coefficients and lower small world and shortest path lengths in the theta and alpha bands compared to the remaining three states. **P* < 0.05, ***P* < 0.01, ****P* < 0.001. The legends were shown below the figure.
